# Epidemiology of enteroaggregative *Escherichia coli* infections and associated outcomes in the MAL-ED birth cohort

**DOI:** 10.1371/journal.pntd.0005798

**Published:** 2017-07-24

**Authors:** Elizabeth T. Rogawski, Richard L. Guerrant, Alexandre Havt, Ila F. N. Lima, Pedro H. Q. S. Medeiros, Jessica C. Seidman, Benjamin J. J. McCormick, Sudhir Babji, Dinesh Hariraju, Ladaporn Bodhidatta, Jasmin Shrestha, Japhat Anania, Athanasia Maro, Amidou Samie, Pablo Peñataro Yori, Shahida Qureshi, Mustafa Mahfuz, Pascal O. Bessong, Margaret N. Kosek, Tahmeed Ahmed, Zulfiqar A. Bhutta, Dennis R. Lang, Michael Gottlieb, Eric R. Houpt, Aldo A. M. Lima, the MAL-ED Network Investigators

**Affiliations:** 1 Department of Public Health Sciences, University of Virginia, Charlottesville, Virginia, United States of America; 2 Division of Infectious Diseases and International Health, University of Virginia, Charlottesville, Virginia, United States of America; 3 Clinical Research Unit and Institute of Biomedicine, Federal University of Ceara, Fortaleza, Brazil; 4 Fogarty International Center, National Institutes of Health, Bethesda, Maryland, United States of America; 5 Division of Gastrointestinal Sciences, Christian Medical College, Vellore, India; 6 Department of Enteric Diseases, Armed Forces Research Institute of Medical Sciences, Bangkok, Thailand; 7 Walter Reed-AFRIMS Research Unit, Nepal, Kathmandu, Nepal; 8 Haydom Global Health Research Center, Haydom Lutheran Hospital, Haydom, Tanzania; 9 Department of Microbiology, University of Venda, Thohoyandou, South Africa; 10 Asociación Benéfica PRISMA, Iquitos, Peru; 11 Department of Paediatrics and Child Health, Aga Khan University, Karachi, Pakistan; 12 Nutrition and Clinical Services Division, International Centre for Diarrhoeal Disease Research, Dhaka, Bangladesh; 13 Department of International Health, Bloomberg School of Public Health, Johns Hopkins University, Baltimore, Maryland, United States of America; 14 Foundation for the National Institutes of Health, Bethesda, Maryland, United States of America; Oxford University Clinical Research Unit, VIET NAM

## Abstract

**Background:**

Enteroaggregative *E*. *coli* (EAEC) have been associated with mildly inflammatory diarrhea in outbreaks and in travelers and have been increasingly recognized as enteric pathogens in young children with and without overt diarrhea. We examined the risk factors for EAEC infections and their associations with environmental enteropathy biomarkers and growth outcomes over the first two years of life in eight low-resource settings of the MAL-ED study.

**Methods:**

EAEC infections were detected by PCR gene probes for *aatA* and *aaiC* virulence traits in 27,094 non-diarrheal surveillance stools and 7,692 diarrheal stools from 2,092 children in the MAL-ED birth cohort. We identified risk factors for EAEC and estimated the associations of EAEC with diarrhea, enteropathy biomarker concentrations, and both short-term (one to three months) and long-term (to two years of age) growth.

**Results:**

Overall, 9,581 samples (27.5%) were positive for EAEC, and almost all children had at least one detection (94.8%) by two years of age. Exclusive breastfeeding, higher enrollment weight, and macrolide use within the preceding 15 days were protective. Although not associated with diarrhea, EAEC infections were weakly associated with biomarkers of intestinal inflammation and more strongly with reduced length at two years of age (LAZ difference associated with high frequency of EAEC detections: -0.30, 95% CI: -0.44, -0.16).

**Conclusions:**

Asymptomatic EAEC infections were common early in life and were associated with linear growth shortfalls. Associations with intestinal inflammation were small in magnitude, but suggest a pathway for the growth impact. Increasing the duration of exclusive breastfeeding may help prevent these potentially inflammatory infections and reduce the long-term impact of early exposure to EAEC.

## Author summary

Enteroaggregative *E*. *coli* (EAEC) are pathogens that infect the intestine and can cause diarrhea. They are also commonly identified among young children in low-resource settings, who can carry the pathogen without symptomatic diarrhea. We examined the risk factors for EAEC infections and their associations with child health outcomes over the first two years of life in eight low-resource settings of the MAL-ED study. EAEC infections were detected using molecular methods in more than 30,000 stools collected from 2,092 children in the MAL-ED study. We identified risk factors for EAEC and estimated the associations of EAEC with diarrhea, markers of intestinal health, and child growth. Almost all children were infected with EAEC at least once by two years of age. Exclusive breastfeeding, higher enrollment weight, and recent macrolide antibiotic use were protective against these infections. Although not associated with diarrhea in these children, EAEC infections were associated with intestinal inflammation and reduced length at two years of age. EAEC may impact child development, even in the absence of diarrhea, by causing intestinal inflammation and impairing child growth.

## Introduction

Enteroaggregative *Escherichia coli* (EAEC) infections have been increasingly recognized as important enteropathogens since their initial discovery by patterns of adherence to HEp-2 cells in *E*. *coli* isolates from Chilean children with diarrhea [1]. EAEC have since been associated with foodborne outbreaks of diarrhea [2], traveler’s diarrhea [3–5], diarrhea in adults with HIV infection [6], endemic diarrhea in cities in the US [7], and variably in healthy adult human volunteers [8,9]. A meta-analysis of 41 studies found EAEC to be significantly associated with acute diarrheal illness among both children and adults in developing regions [10]. However, because EAEC are also a highly common infection among children without overt diarrhea in low-resource settings, they have not been found to be a major cause of diarrhea in some endemic settings [11,12]. Regardless, EAEC, independent of diarrheal symptoms, have been associated with other poor health outcomes in children, such as growth failure [13] and mild to moderate intestinal inflammation [5,13,14].

The genetic determinants and biological mechanism for the virulence of EAEC have been described by a complex array of interacting traits that reside on both the chromosome and plasmid in the organism [15,16]. As presently defined, EAEC are heterogeneous with respect to virulence gene content. The *aggR* trait on the plasmid is a common and well-characterized EAEC gene [17] that regulates many virulence traits, including chromosomal *aaiC*, which is in the gene cluster *aaiA-Y* that encodes the type VI secretion system, as well as plasmid-borne *aatA*, which encodes an ABC transporter. In addition, the flagellin of EAEC strain 042 has been shown to trigger inflammation via TLR5 signaling [18,19]. Murine models have helped determine the impact of these virulence genes by providing evidence that EAEC can cause inflammation, enteropathy, and growth shortfalls among mice with dietary protein deficiency [20,21], and even diarrhea among mice with dietary zinc deficiency [22].

Increasing evidence suggests that enteric infections, especially common pathogens like EAEC, may play an important role in morbidity due to enteric disease, beyond symptomatic diarrhea [23]. While mortality from diarrheal diseases has been dramatically reduced to less than half a million deaths per year [24], more than a quarter of the world’s children are moderately or severely stunted [25]. Because improved feeding does not eliminate growth shortfalls in low-resource areas where inadequate water and sanitation and heavy burdens of enteric infections are common [26,27], enteric infections and sub-clinical environmental enteropathy likely also contribute to poor child growth outcomes [28,29].

We characterized the epidemiology and impact of EAEC infections among children in the first two years of life in eight low-resource settings of the Etiology, Risk Factors, and Interactions of Enteric Infections and Malnutrition and the Consequences for Child Health and Development Project (MAL-ED) study. With twice-weekly active surveillance from near birth to two years of age, the MAL-ED study provides a unique opportunity to assess the impact of both clinical and subclinical enteric infections on early-life growth and development. We examined risk factors for EAEC infections and their associations with diarrhea, environmental enteropathy biomarkers, and growth outcomes over the first two years of life.

## Methods

The study design and methods of the MAL-ED study have been extensively described [30]. Briefly, children were enrolled within 17 days of birth and followed until two years of age at eight sites: Dhaka, Bangladesh (BGD), Vellore, India (INV), Bhaktapur, Nepal (NEB), Naushahro Feroze, Pakistan (PKN), Fortaleza, Brazil (BRF), Loreto, Peru (PEL), Venda, South Africa (SAV), and Haydom, Tanzania (TZH). Non-diarrheal surveillance stool samples were collected monthly and diarrheal stool samples were collected during 94% of diarrhea episodes identified by active surveillance at twice weekly home visits. Diarrhea was defined as maternal report of three or more loose stools in 24 hours or one stool with visible blood [31]. Monthly surveillance stool samples in the first year of life, quarterly stool samples in the second year of life, and all diarrheal stool samples were tested for more than 50 enteropathogens [32] and stool biomarkers of environmental enteropathy: α-1-antitrypsin (ALA), myeloperoxidase (MPO), and neopterin (NEO) [33]. For EAEC specifically, we picked and pooled five lactose-fermenting colonies resembling *E*. *coli*, and characterized them for virulence genes using a multiplex polymerase chain reaction (PCR) assay. Presence of the enteroaggregative *E*. *coli* pathotype was defined by amplification of either the *aatA* or *aaiC* virulence genes (or both) [32], such that detected EAEC were heterogeneous with respect to virulence gene content. Results were consistent when requiring the presence of both *aatA* and *aaiC* to define EAEC. We included all stool samples that were tested for EAEC in this analysis even if they were not tested for the full suite of other pathogens.

Fieldworkers also collected information on other illnesses, medicines, and feeding practices at home visits. Sociodemographic information was collected by questionnaire biannually and summarized using a construct of access to improved water and sanitation (as defined by WHO guidelines [34]), wealth measured by eight assets, years of maternal education, and average monthly household income (Water, Assets, Maternal education, and Income, WAMI) [35]. Plasma α-1-acid glycoprotein (AGP), a marker of systemic inflammation, was measured at 7, 15, and 24 months. Urinary lactulose:mannitol excretion ratios were measured at 3, 6, 9 and 15 months and converted into a sample-based z-score (LMZ) using the Fortaleza, Brazil cohort as the internal reference population [36]. Weight and length were measured monthly and converted into weight-for-age (WAZ) and length-for-age (LAZ) z-scores using the 2006 WHO child growth standards [37]. Length measurements from Pakistan were excluded due to measurement quality concerns.

### Ethics statement

The study was approved by the Institutional Review Board for Health Sciences Research, University of Virginia, USA as well as the respective governmental, local institutional, and collaborating institutional ethical review boards at each site: Ethical Review Committee, ICDDR,B (BGD); Committee for Ethics in Research, Universidade Federal do Ceara; National Ethical Research Committee, Health Ministry, Council of National Health (BRF); Institutional Review Board, Christian Medical College, Vellore; Health Ministry Screening Committee, Indian Council of Medical Research (INV); Institutional Review Board, Institute of Medicine, Tribhuvan University; Ethical Review Board, Nepal Health Research Council; Institutional Review Board, Walter Reed Army Institute of Research (NEB); Institutional Review Board, Johns Hopkins University; PRISMA Ethics Committee; Health Ministry, Loreto (PEL); Ethical Review Committee, Aga Khan University (PKN); Health, Safety and Research Ethics Committee, University of Venda; Department of Health and Social Development, Limpopo Provincial Government (SAV); Medical Research Coordinating Committee, National Institute for Medical Research; Chief Medical Officer, Ministry of Health and Social Welfare (TZH). Informed written consent was obtained from the parent or guardian of each participating child on their behalf.

### Data analysis

We identified risk factors for EAEC detection in surveillance stools using log-binomial regression with general estimating equations (GEE) and robust variance to account for correlation between stools within children, adjusting for site and a restricted quadratic spline [38] for age. Variables were assessed individually in this model and were included in the multivariable model if statistically significant (p<0.05). We estimated the association between EAEC and diarrheal versus non-diarrheal stools using Poisson regression with the robust variance estimator to estimate risk ratios [39] since log-binomial models did not converge, adjusting for the age spline, site, the interaction between age and site, and antibiotic use within the preceding 15 days.

We then estimated the association between EAEC detection and stool biomarker concentrations (ALA, MPO, and NEO) on the logarithmic scale in the same stool using multivariable linear regression with GEE and robust variance to account for correlation between stools within children. We also estimated the association of EAEC detection with serum and urine biomarkers (AGP and LMZ, respectively) measured in the same month as the stool collection. Because *Campylobacter* was the most common pathogen detected in stools and has been previously shown to be associated with intestinal inflammation in the MAL-ED cohort [40,41], we assessed potential interactions between the effects of EAEC and *Campylobacter* on MPO by including an interaction term between presence of EAEC and *Campylobacter*. All estimates were adjusted for site, the age spline, sex, WAMI, percent exclusive breastfeeding in previous month, contemporary presence of *Campylobacter* in the stool sample, and a qualitative description of stool consistency (for stool biomarkers only).

Finally, we estimated the association between EAEC detection and short-term and long-term growth using multivariable linear regression. Short-term growth was defined by the change in WAZ and LAZ over both the one and three months following each monthly stool collection. We compared differences in short-term growth velocity between children who had surveillance stools with and without EAEC detection, using GEE and adjusting for site, age, sex, WAMI, percent exclusive breastfeeding in the exposure month, and detection of *Campylobacter* in the stool.

We further assessed the interaction between MPO levels and EAEC positivity to explore the role of intestinal inflammation in the potential effect of EAEC on short-term growth impairment. In the adjusted short-term growth models examining WAZ and LAZ velocity over the one and three months following EAEC testing, we estimated the additive interaction effect of EAEC detection and high MPO concentration in the same stool using an interaction term. High MPO was defined as an MPO concentration in the highest quartile on the logarithmic scale among all non-diarrheal stools collected at that child’s site and 3-month age period. Values defining high MPO (range: 2,515–33,190 ng/mL) were higher than previous reports from non-tropical settings (<2,000 ng/mL) [42].

Effects on long-term growth were then estimated as the total difference in size at two years of age as a function of the percent surveillance stools positive for EAEC. The long-term model was adjusted for the WAZ and LAZ measurements at enrollment (within 17 days of birth), site, sex, WAMI, the age at which exclusive breastfeeding first stopped, and the percent surveillance stools positive for *Campylobacter* in the first 2 years of life. Adjusting for the same covariates, we assessed the potential synergistic interaction between the effects of EAEC and *Campylobacter* on growth at 2 years given that both have been associated with gut inflammation, by including an interaction term between an indicator for a high frequency of detection (at least 50% surveillance stools positive) of EAEC and an indicator for a high frequency of detection of *Campylobacter*. We also repeated the model described above, but focused on EAEC detections in specific age periods (1–6, 7–12, and 15–24 months) and growth outcomes at 2 years to assess if there were specific age periods of susceptibility.

## Results

We included 27,094 non-diarrheal surveillance stools and 7,692 diarrheal stools that were tested for EAEC from 2,092 children who each contributed at least one stool sample in the MAL-ED birth cohort. 1,736 (83.0%) of these children were followed to two years of age. Overall, 9,581 samples (27.5%) were positive for EAEC; *aatA* was detected in 41.6% (n = 3,982) of EAEC-positive stool samples, *aaiC* was detected in 31.4% (n = 3,007), and both genes together were detected in 27.1% (n = 2,592).

**Fig 1:**
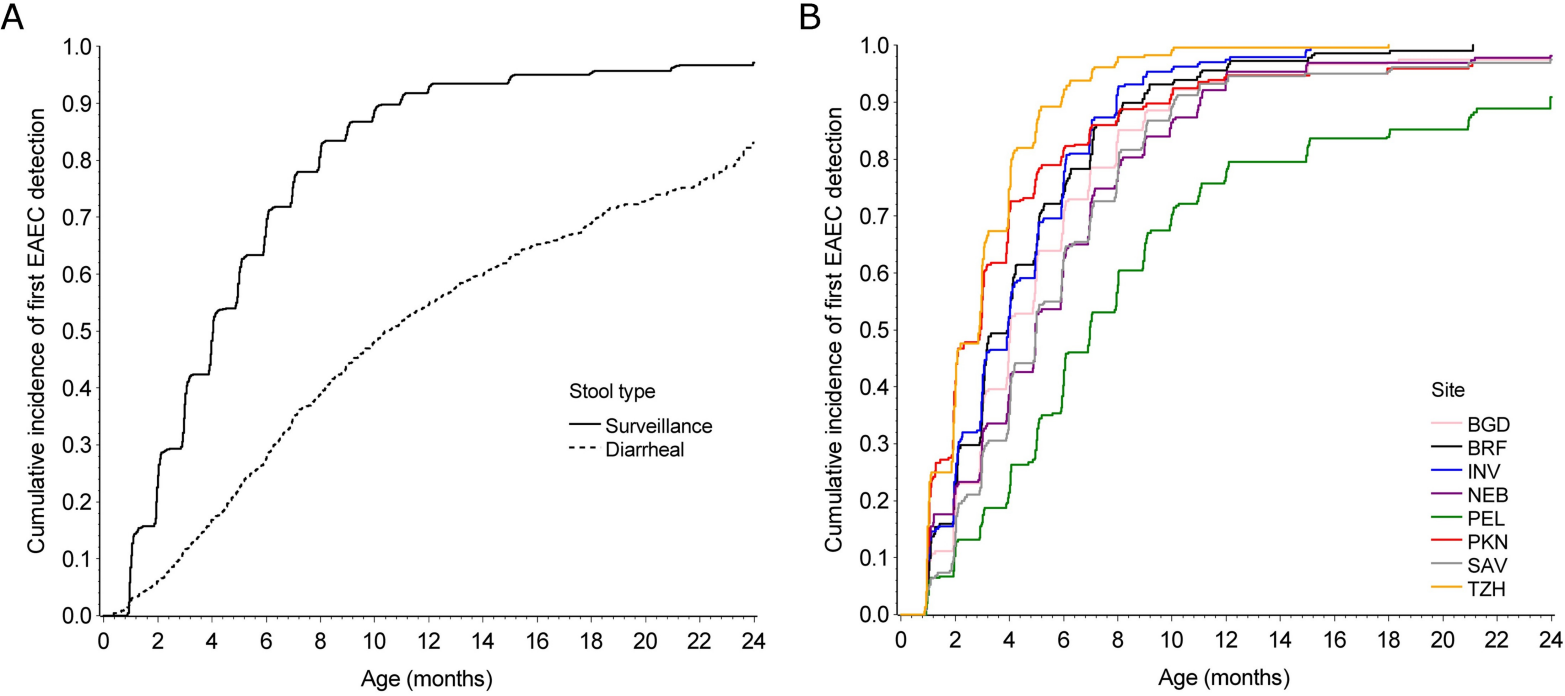
Incidence of EAEC. Cumulative incidence of first EAEC detection in A) surveillance and diarrheal stools at all sites and B) surveillance stools by site among 2,092 children with at least one stool sample in the MAL-ED birth cohort. BGD–Dhaka, Bangladesh; BRF–Fortaleza, Brazil; INV–Vellore, India; NEB–Bhaktapur, Nepal; PEL–Loreto, Peru; PKN–Naushahro Feroze, Pakistan; SAV–Venda, South Africa; TZH–Haydom, Tanzania.

EAEC was detected in at least one stool for almost all children (n = 1,983, 94.8%) by two years of age, and detection in a surveillance stools preceded detection in a diarrheal stool for 82.2% (n = 1,631) of these children (Fig 1A). The median time to first detection in surveillance stools was 4.0 months and ranged from 2.9 months in Tanzania to 7.0 months in Peru (Fig 1B). Repeated detections among children were common, with a range of 0–15 detections per child when including surveillance and diarrheal stools. The median number of detections in surveillance stools among children who completed two years of follow-up was 2 in Peru, 3 in South Africa, and 4 or 5 at all other sites.

### Risk factors

Because of the near ubiquity of EAEC detection in these study sites, few factors were identified that were associated with EAEC detection in surveillance stools. Enrollment weight, exclusive breastfeeding, and recent macrolide use were the only protective factors in the multivariable analysis, and only the associations with the latter two had a substantial magnitude of effect (Table 1). Socioeconomic status (WAMI) was weakly protective, but the association was not statistically significant. Macrolide use in the past 15 days, but not cephalosporin use nor any other antibiotic use, was associated with a reduction in EAEC detection. However, macrolide use in the past 16–30 days was not protective (RR: 0.94, 95% CI: 0.85, 1.05). This short-term only effect of macrolide use was consistent across all sites and ages.

**Table 1:** Risk factors for EAEC detection in monthly surveillance stools among 2,091 children in the MAL-ED cohort with at least one surveillance stool.

	Semi-univariable*	Multivariable^†^
Risk factor	Risk ratio (95% CI)	Risk ratio (95% CI)
*Child characteristics*		
Female (vs. male)	0.99 (0.96, 1.04)	
Enrollment weight (per 1 weight-for-age z-score)	0.97 (0.95, 0.99)	0.97 (0.95, 0.99)
Percent days exclusively breastfed in last month^‡^	0.72 (0.65, 0.79)	0.72 (0.65, 0.79)
WAZ (per 1 z-score)^§^	0.98 (0.96, 1.00)	
LAZ (per 1 z-score)^§^**	0.99 (0.97, 1.01)	
WLZ (per 1 z-score)^§^**	1.00 (0.98, 1.02)	
Any antibiotic use in past 15 days	0.99 (0.94, 1.04)	
Macrolide use in past 15 days	0.77 (0.68, 0.86)	0.76 (0.68, 0.85)
*Sociodemographic*		
Socioeconomic score (WAMI [35]; per 0.5 units)	0.94 (0.87, 1.01)	
Household income at or above site-specific median income	0.96 (0.92, 1.01)	
Maternal age (per 5 years)	0.99 (0.96, 1.02)	
Maternal education (6 or more years completed)	0.99 (0.94, 1.04)	
Mother married	0.92 (0.85, 1.00)	
Child has siblings	1.03 (0.98, 1.07)	
Mean number of people per room in the household (per 1 unit)	1.01 (0.99, 1.02)	
*Water*, *sanitation*, *and hygiene*		
Improved drinking water [34] (vs. unimproved)	1.06 (0.96, 1.17)	
Time to access water (>10 minutes)	1.03 (0.96, 1.11)	
Treated water (vs. untreated)	1.02 (0.95, 1.10)	
Improved sanitation [34] (vs. unimproved)	0.95 (0.89, 1.02)	
Share toilet facility	1.06 (0.99, 1.13)	
*Environmental*		
Dirt floor	1.03 (0.97, 1.10)	
Household owns cows	0.95 (0.88, 1.03)	
Household owns chickens	1.00 (0.93, 1.08)	

*Adjusted for site and age only (using restricted quadratic splines)

†Adjusted for site, age, and all other variables with estimates in this column

‡ Included as a continuous variable; risk ratio is scaled for the comparison of exclusive breastfeeding on all days in previous month to exclusive breastfeeding on no days in the previous month

§At most recent measurement prior to stool collection. WAZ–weight-for-age z-score; LAZ–length-for-age z-score; WLZ–weight-for-length z-score.

**Excluding Pakistan.

### EAEC and diarrhea

Adjusting for age, site, and their interaction, EAEC was not associated with diarrhea and was found significantly more often in surveillance stools compared to diarrheal stools (RR: 0.86, 95% CI: 0.82, 0.90). This association remained when adjusting for recent antibiotic use and specifically macrolide use, as well as if restricted to only those children with no antibiotic use in the past 30 days. Similarly, presence of EAEC in stools was not associated with persistent diarrhea (duration of 14 days or more; RR: 0.93, 95% CI: 0.73, 1.18) compared to non-diarrheal stools.

### Association with markers of environmental enteropathy

EAEC detection was associated with higher contemporary concentrations of MPO (MPO 0.14 ln(ng/mL), 95% CI: 0.11, 0.18 higher in the presence of EAEC), a marker of intestinal inflammation, at all sites (Table 2). It was also associated with higher levels of ALA (permeability) and NEO (intestinal inflammation) overall, with some variation across sites. However, the magnitudes of these associations were very small (1.15 ng/mL difference in MPO) compared to the range of observed concentrations in the study (MPO interquartile range: 2,050–12,920 ng/mL). In addition, EAEC was not associated with AGP, a marker of systemic inflammation, nor the lactulose-mannitol ratio, a marker of intestinal permeability, measured during the same month as the stool collection.

**Table 2:** Associations between EAEC detection and markers of inflammation and gut permeability in surveillance and diarrheal stools among 2,076 children in the MAL-ED cohort with at least one biomarker measurement.

Site	MPO concentration* (95% CI) N = 24,622	ALA concentration* (95% CI) N = 24,622	NEO concentration* (95% CI) N = 24,769	AGP concentration* (95% CI) N = 4,760	LMZ* (95% CI) N = 7,285
BGD	0.12 (0.01, 0.23)	0.02 (-0.07, 0.11)	0.04 (-0.07, 0.16)	4.05 (-3.59, 11.69)	-0.02 (-0.16, 0.13)
BRF	0.22 (0.10, 0.34)	-0.03 (-0.11, 0.06)	0.08 (-0.01, 0.17)	-2.48 (-10.54, 5.59)	0.07 (-0.17, 0.30)
INV	0.21 (0.12, 0.29)	0.07 (-0.01, 0.14)	0.10 (0.03, 0.18)	-4.83 (-11.18, 1.53)	-0.13 (-0.29, 0.03)
NEB	0.08 (-0.01, 0.17)	-0.05 (-0.13, 0.03)	0.05 (-0.01, 0.11)	3.35 (-5.30, 12.00)	-0.14 (-0.29, 0.01)
PEL	0.21 (0.10, 0.32)	0.07 (-0.04, 0.17)	0.08 (-0.01, 0.16)	-2.37 (-13.52, 8.77)	-0.18 (-0.32, -0.04)
PKN	0.16 (0.05, 0.26)	0.14 (0.04, 0.25)	-0.19 (-0.30, -0.08)	0.32 (-7.24, 7.87)	-0.03 (-0.21, 0.16)
SAV	0.07 (-0.03, 0.16)	0.14 (0.04, 0.24)	0.10 (0.01, 0.19)	5.87 (-6.53, 18.27)	0.22 (-0.02, 0.47)
TZH	0.12 (0.02, 0.21)	0.1 (0.01, 0.18)	0.18 (0.06, 0.30)	1.70 (-9.24, 12.64)	-0.02 (-0.17, 0.13)
All	0.14 (0.11, 0.18)	0.06 (0.03, 0.09)	0.06 (0.02, 0.09)	0.52 (-2.63, 3.68)	-0.05 (-0.12, 0.02)

*Difference in concentration comparing stools with and without EAEC detection, adjusted for site, age, sex, WAMI, percent exclusive breastfeeding, presence of *Campylobacter* in stool sample, and stool consistency (MPO, ALA, NEO models only).

LMZ: Urinary lactulose:mannitol excretion ratio z-score measured at 3, 6, 9, and 15 months using the BRF cohort as the internal reference population

ALA: α-1-antitrypsin (ln(mg/g))

MPO: myeloperoxidase (ln(ng/mL))

NEO: neopterin (ln(nmol/L))

AGP: α-1-acid glycoprotein (mg/dL) measured at 7, 15, and 24 months.

BGD–Dhaka, Bangladesh

BRF–Fortaleza, Brazil

INV–Vellore, India

NEB–Bhaktapur, Nepal

PEL–Loreto, Peru

PKN–Naushahro Feroze

Pakistan

SAV–Venda, South Africa

TZH–Haydom, Tanzania

EAEC was associated with elevated MPO independently of *Campylobacter*, but their combined effect on MPO was less than additive when both pathogens were present. Detection of EAEC alone was associated with an adjusted 0.17 (95% CI: 0.13, 0.21) higher ln(MPO) concentration, *Campylobacter* alone was associated with an adjusted 0.19 (95% CI: 0.15, 0.24) higher concentration, and the detection of both pathogens was associated with an adjusted 0.27 (95% CI: 0.21, 0.34) higher concentration.

### Effects of EAEC infection on growth

**Fig 2:**
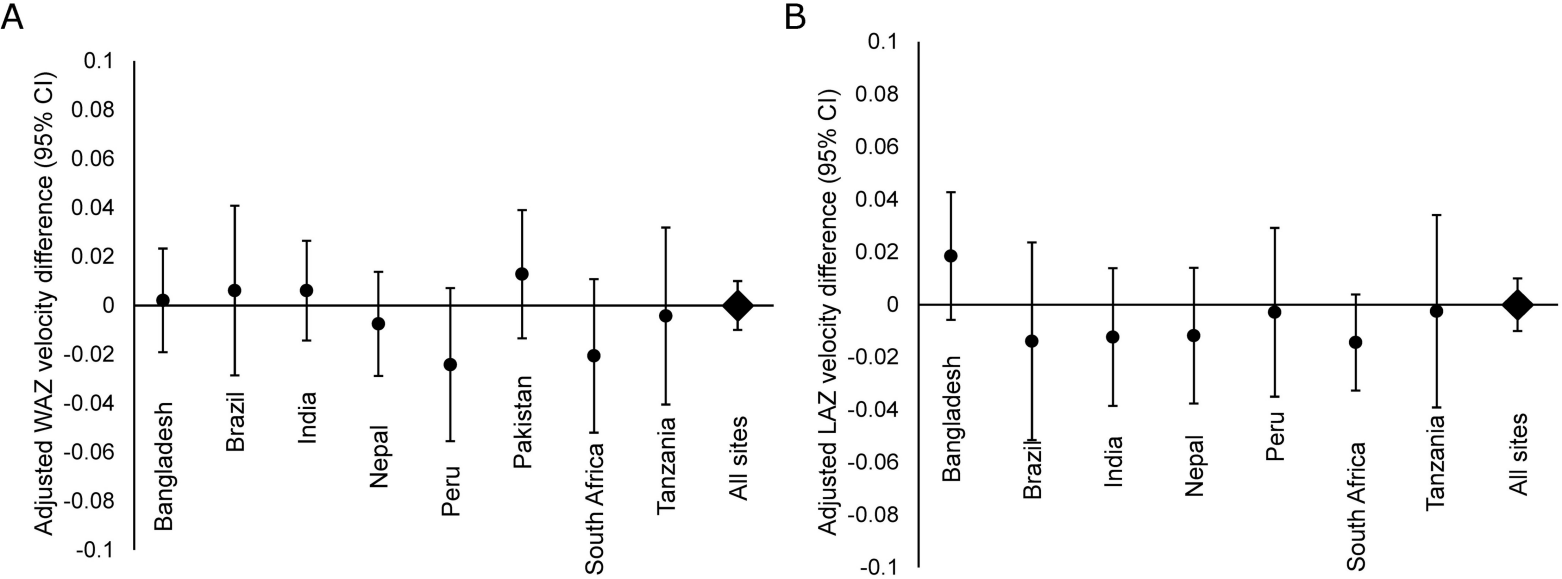
Short-term growth. Adjusted site-specific associations between EAEC detection in monthly surveillance stools and A) weight-for-age z-score (WAZ) velocity and B) length-for-age z-score (LAZ) velocity over the subsequent month among 2,050 children in the MAL-ED cohort with at least one surveillance stool and at least one month of complete anthropometric measurements and testing for EAEC and *Campylobacter*.

Detection of EAEC was not associated with short term differences in growth velocity in both the one and three months following each monthly stool collection overall or at any site (Fig 2). Furthermore, there was no evidence of an interaction between EAEC detection and MPO in the same stool (*p* for interaction: 0.9 and 0.5 for 1-month WAZ and LAZ velocity, respectively); concurrent detection of EAEC and a high level of MPO were also not associated with short-term WAZ and LAZ velocity.

**Fig 3:**
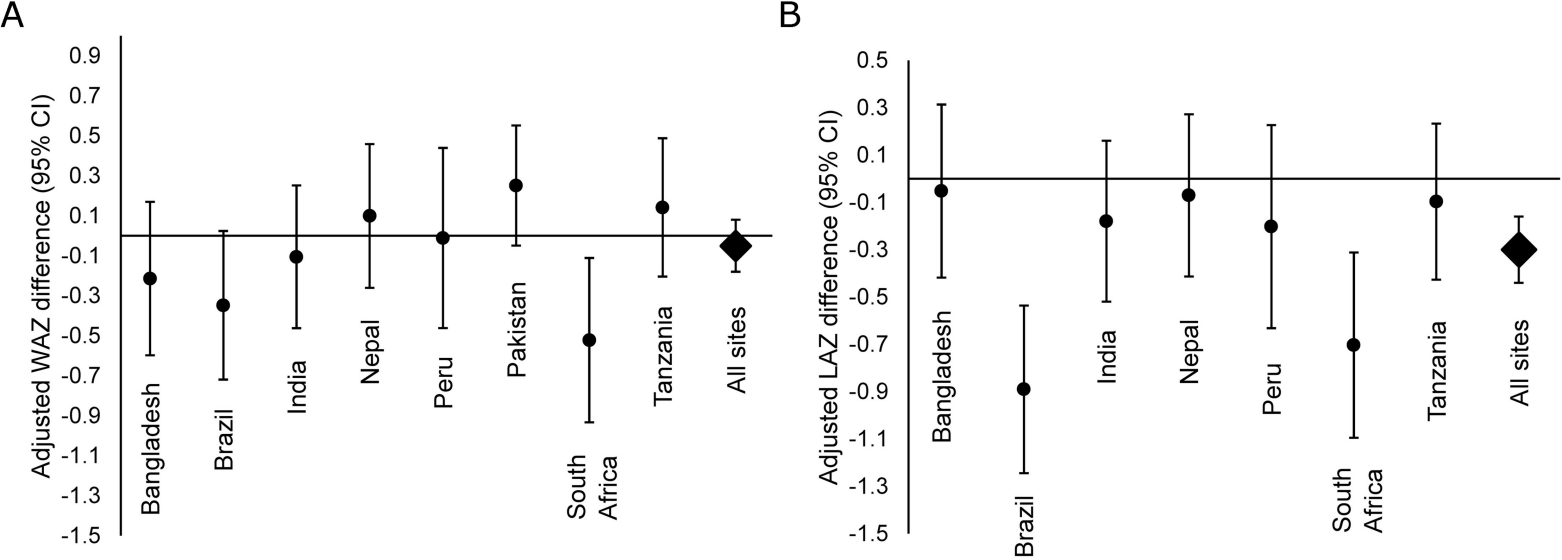
Long-term growth. Adjusted site-specific association between EAEC detection in monthly surveillance stools and A: weight-for-age z-score (WAZ) and B: length-for-age z-score (LAZ) at two years of age among 1,727 children in the MAL-ED cohort who had anthropometric measurements at two years. Estimates are the z-score difference associated with a high frequency of EAEC detection compared to a low frequency of EAEC detection. Definitions for high and low frequency are based on the 10^th^ and 90^th^ percentiles of stool positivity in the cohort. Low: ≤11% of surveillance stools positive for EAEC; high: ≥50% of surveillance stools positive for EAEC.

Over the course of the first two years of life, there was no difference at 2 years in WAZ (overall difference: -0.05, 95% CI: -0.18, 0.08) associated with a linear increase in EAEC stool positivity (Fig 3). In contrast, more detections of EAEC were associated with significant decrements in LAZ (Fig 3). The difference in LAZ at 2 years of age between a child at the 90^th^ percentile of EAEC stool positivity from 0–2 years (50% stools positive) compared to a child at the 10^th^ percentile for EAEC stool positivity (11% stools positive) was -0.30 LAZ (95% CI: -0.44, -0.16). Among site-specific estimates, this association was greatest in Brazil (LAZ difference at 2 years: -0.89, 95% CI: -1.24, -0.54) and South Africa (LAZ difference at 2 years: -0.70, 95% CI: -1.09, -0.31).

There was evidence for an antagonistic interaction between high frequency of EAEC detection (at least 50% of stools positive) and high frequency of *Campylobacter* detection on the adjusted LAZ difference at two years, such that high detection of both pathogens was associated with a similar decrement in LAZ (-0.29, 95% CI: -0.74, 0.15) as that for high detection of either pathogen alone (EAEC: -0.38, 95% CI: -0.54, -0.22; *Campylobacter*: -0.29, 95% CI: -0.43, -0.14).

A high frequency of EAEC detection during only one of the periods 1–6 months, 7–12 months, and 15–24 months was not associated with LAZ decrements, whereas high frequency of detection in any two of the three time periods was associated with small non-significant length decrements, and high frequency of detection in all three time periods was associated with the largest length decrements (Table 3). There were no additional differences in growth between children who had at least one detection of EAEC in a diarrheal stool compared to children who did not after accounting for EAEC detection in surveillance stools (Table 3).

**Table 3:** Effects of EAEC detection in monthly surveillance stools on weight (WAZ) and length (LAZ) attainment at 2 years of age among 1,727 children in the MAL-ED cohort with anthropometric measurements at 2 years.

High frequency of EAEC detection in age period*	No. exposed (%) N = 1736	WAZ difference at 2 years (95% CI)^†^	No. exposed (%) N = 1487	LAZ difference at 2 years (95% CI) ^†^
1–6 mo. only	212 (12.2)	0.07 (-0.08, 0.21)	159 (10.7)	0.02 (-0.14, 0.17)
7–12 mo. only	261 (15.0)	0.05 (-0.08, 0.18)	236 (15.9)	-0.01 (-0.15, 0.12)
15–24 mo. only	203 (11.7)	0.08 (-0.06, 0.22)	181 (12.2)	0.01 (-0.13, 0.15)
1–6 mo. & 7–12 mo.	104 (6.0)	0.13 (-0.06, 0.32)	76 (5.1)	-0.25 (-0.46, -0.04)
1–6 mo. & 15–24 mo.	72 (4.1)	0.01 (-0.21, 0.23)	52 (3.5)	-0.18 (-0.43, 0.07)
7–12 mo. & 15–24 mo.	114 (6.6)	-0.10 (-0.28, 0.08)	106 (7.1)	-0.20 (-0.39, -0.02)
All three periods	43 (2.5)	-0.45 (-0.73, -0.17)	35 (2.4)	-0.59 (-0.89, -0.29)
Any EAEC diarrhea	846 (48.7)	0.05 (-0.05, 0.14)	640 (43.0)	0.07 (-0.02, 0.18)

*At least 50% of surveillance stools in the period were positive for EAEC.

†Adjusted for site, anthropometric measurement at enrollment, sex, WAMI, age at stopping exclusive breastfeeding, percent surveillance stools positive for *Campylobacter* in the first 2 years of life.

All LAZ estimates exclude Pakistan

## Discussion

We identified widespread acquisition of EAEC within the first few months of life across diverse settings in South Asia, South America, and Africa. In all sites except Peru, EAEC was detected at least once by two years of age in more than 90% of enrolled children. Slightly lower detection of EAEC in Peru may be due to the relatively high rates of macrolide use observed at this site in MAL-ED [43]. A high prevalence of EAEC in children with and without diarrhea was also found in the seven-site Global Enteric Multicenter Study, a prospective matched case-control study of moderate-to-severe diarrhea [11]. There was no evidence in either study that EAEC was a major cause of diarrhea of any duration.

Few risk factors for EAEC were identified in this analysis, and surprisingly, components of socioeconomic status and our index, the WAMI, were not consistently protective. Only exclusive breastfeeding, enrollment weight, and recent macrolide use were associated with reduced EAEC detections. Exclusive breastfeeding is protective against enteric infections through multiple pathways, including limits on environmental exposure through contaminated food and water and directly through antimicrobial factors like lactoferrin and antibodies present in breastmilk [44]. The percent days of exclusive breastfeeding accounts for temporary cessation and return to exclusivity, and the protective association of this construct emphasizes that the age of first stopping exclusivity may be less important than the practice of exclusive breastfeeding itself, which may occur in multiple episodes [45]. The association of EAEC infections with lower enrollment weight is consistent with the increased susceptibility of malnourished mice to EAEC infection compared to well-nourished mice [20].

Antimicrobial resistance is a common feature of EAEC [46–48], and at least one EAEC-specific resistance island has been characterized [49]. This island does not contain resistance genes for macrolides, which may explain the protective association with macrolide use (unlike either cephalosporin or any class of antibiotic use). The specificity of protection by macrolides may provide EAEC with a competitive advantage over other enteropathogens since non-macrolide antibiotic use was highly frequent at many of the MAL-ED sites [43]. Further characterization of the antimicrobial resistance of these isolates will be necessary to confirm this hypothesis.

Because only recent macrolide use was protective against EAEC infections, clearance of EAEC may be incomplete or more likely, reinfection with EAEC occurs quickly. In addition, alterations of the microbiota by macrolides could increase susceptibility to later EAEC infections, as is evident in murine infections [21]. Therefore, antibiotic use to clear EAEC infections is likely not justified; however, increasing the duration of exclusive breastfeeding (even if in separated episodes) may delay the acquisition of these common, potentially inflammatory infections.

EAEC detection was associated with markers of intestinal inflammation, most strongly with increased fecal MPO. While the magnitudes of the associations were small relative to the range of observed concentrations, the increase in average levels of fecal MPO associated with EAEC [0.17 ln(ng/ml)] was comparable to that seen with *Campylobacter* infections [0.19 ln(ng/ml)], which is a recognized cause of inflammatory enteritis [50,51]. EAEC has been previously associated with markers of inflammation, specifically with lactoferrin [52] and the proinflammatory cytokines interleukin (IL)-1b [14] and IL-8 [13,14,53]. The relevance of elevated intestinal inflammation to potential systemic inflammation associated with EAEC is not clear; there was no evidence that EAEC was associated with elevated AGP, a marker of systemic inflammation, though we note AGP was tested less frequently in this study and could have captured highly acute responses that may not have been temporarily coincident with stool sampling.

The association between EAEC and intestinal inflammation suggests a potential mechanism for the observed association between EAEC and growth. Intestinal inflammation [54] and specifically higher levels of fecal MPO [55–57], have been associated with poor linear growth among children in Brazil, Bangladesh, and the Gambia. However, because the magnitudes of association with inflammatory biomarkers were very small, this pathway may not be a major contributor to the overall growth impact, or equally, the biomarkers measured may be suboptimal markers.

EAEC was associated with substantial decrements in LAZ at two years of age, and the magnitude of this association was similar to that reported for *Campylobacter* in MAL-ED [40]. However, the effects were less than additive, such that a high frequency of detection of both pathogens was associated with similar decrements as those associated with either pathogen alone. In contrast, EAEC was not associated with WAZ. The lack of association of EAEC with short-term growth velocity of either weight or length and the fact that the greatest impact of EAEC occurred among children with the highest frequency of detection during the first 2 years of life suggest that repeated high rates of exposure to EAEC prolonged over many months is necessary for the manifestation of overall length decrements observed at two years of age. Continual carriage and/or re-infection with a pathogen that is ubiquitous in the environment may limit the possibility for catch-up growth resulting in consistent linear shortfalls in the longer-term.

This analysis provides a comprehensive longitudinal assessment of EAEC infections in early life across diverse low-resource settings, drawing on a large number of stool collections, biomarker assessments, and repeated anthropometric measurements. The study was limited by the potentially suboptimal assessment of pathogenic EAEC since the virulence genes for EAEC are not well understood [58], and there may have been differences in strain variability across sites. Our gene probes, *aatA* and *aaiC*, were chosen as characteristic plasmid and chromosomal traits of EAEC, respectively [59], and may not be perfectly discriminating for pathogenic EAEC. Genetic probes generally associate with laboratory phenotypes, not necessarily clinical disease [49,60]. In a study of children in Mali, *aatA* and *aaiC* were not associated with diarrhea when considering presence of either gene alone or in combination [61]. Furthermore, EAEC is able to acquire additional virulence genes that could increase its pathogenicity, such as the acquisition of Stx2 phage (a characteristic of enterohemorrhagic *E*. *coli*) in a German outbreak of EAEC-associated gastroenteritis [62]. The potential inability to distinguish pathogenic versus non-pathogenic EAEC may contribute to the weak associations observed between EAEC, inflammatory biomarkers, and short-term growth velocity.

In conclusion, we found that EAEC infections were very common in the eight MAL-ED sites over the first two years of life. While often acutely subclinical, repeated EAEC detections were associated with longer-term linear growth deficits. Further work is needed better quantify the contribution of intestinal inflammation caused by EAEC to impaired growth. Refining our understanding of virulence traits may further help elucidate mechanisms of pathogenesis as well as the potential for vaccine-mediated or other approaches to control these increasingly recognized enteric pathogens. Because these infections may cause lasting consequences in terms of environmental enteropathy and relate to child growth deficits, a better understanding of the mechanisms involved and relevant biomarkers are critical to developing targeted interventions to prevent these consequences for the world’s poorest children.
